# Keeping mitochondria happy – benefits of a pore choice in acute pancreatitis

**DOI:** 10.1113/JP279116

**Published:** 2019-11-28

**Authors:** David N. Criddle

**Affiliations:** ^1^ Department of Cellular & Molecular Physiology, Institute of Translational Medicine University of Liverpool UK

**Keywords:** acute pancreatitis, cyclophilin D, mitochondrial transition pore

Mitochondrial dysfunction is a key feature of multiple diseases and thus protection of this organelle is an important therapeutic objective. The pancreatic acinar cell, which synthesises and stores digestive enzyme precursors, is the most abundant cell type in pancreatic tissue and considered to be the primary site of acute pancreatitis (AP) initiation. Early investigations at the University of Liverpool and by others discovered that precipitants of AP, including bile acids and alcohol non‐oxidative metabolites, disrupt calcium signalling in acinar cells leading to toxicity. Sustained cytosolic calcium elevations raise mitochondrial matrix calcium, triggering the opening of the mitochondrial permeability transition pore (MPTP), which results in a loss of membrane potential and ATP production vital for cellular processes (Criddle *et al*. 2006; Mukherjee *et al*. 2016) (Fig. 1). The prime consequence of pancreatic mitochondrial dysfunction in AP is necrotic cell death, the extent of which is a major determinant of clinical outcome. Subsequent studies have shown that calcium‐dependent mitochondrial dysfunction in response to AP precipitants also occurs in ductal cells, widening the cast of players implicated in the development of AP (Hegyi & Petersen, 2013). There is currently no specific therapy for the disease and protection of mitochondria by MPTP inhibition is considered a promising therapeutic approach.

**Figure 1:**
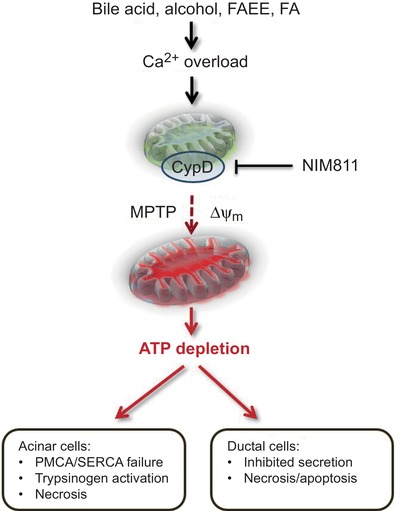
Protective effects of NIM811 in acute pancreatitis The cyclophilin D (CypD) inhibitor NIM811 prevents formation of the mitochondrial permeability transition pore (MPTP) that is triggered by a rise in mitochondrial matrix Ca^2+^ concentration. Precipitants of acute pancreatitis (AP) such as bile acids and alcohol non‐oxidative metabolites – fatty acid ethyl esters (FAEE) and fatty acids (FA) – elicit sustained elevations of cytosolic Ca^2+^ in pancreatic acinar and ductal cells that overload mitochondria inducing MPTP formation, loss of membrane potential and ATP production. NIM811 therefore protects against the damaging consequences of aberrant Ca^2+^ signalling and loss of energy provision, maintaining normal physiological function in acinar and ductal cells, thereby ameliorating AP. PMCA, plasma membrane Ca^2+^‐ATPase; SERCA, sarco/endoplasmic reticulum Ca^2+^‐ATPase.

The precise composition of the MPTP, however, remains controversial and is the subject of vigorous debate. Since discovery of calcium‐dependent permeability transition in mitochondria, many detailed studies have been performed to establish its core components, involving genetic manipulation of candidate pore proteins including the voltage‐dependent anion channel, adenine nucleotide transporter and ATP synthase. Although no firm consensus has yet been reached, it is widely recognised that cyclophilin D is a key modulator of MPTP opening and a prime target for drug development in many pathologies. With respect to AP, genetic deletion and pharmacological inhibition of cyclophilin D were shown to be beneficial in multiple models that reflect different aetiologies. Thus *in vivo* pathological changes in response to bile acid ductal infusion (TLCS‐AP), non‐oxidative ethanol metabolites generated by fat/alcohol administration (FAEE‐AP) (Huang *et al*. 2014) and caerulein hyperstimulation were protected against by treatment with the archetypal cyclophilin D inhibitor cyclosporine A, the newer derivative Debio 025 (Alisporivir; Debiopharm) and cyclophilin D knockout (*ppif^−/−^*) (Mukherjee *et al*. 2016).

However, a successful translation of MPTP inhibitors to the clinic for treatment of AP has not yet been forthcoming. A licensed drug, cyclosporine A, is not considered appropriate for AP therapy due to its immunosuppressant action via calcineurin inhibition. Furthermore, despite promising early preclinical results, its analogue Alisporivir has not progressed to clinical development for AP treatment. Although in a recent clinical trial it was found to be effective as combination therapy against chronic hepatitis C viral (HCV) infection, serious side effects were reported. In addition, an MPTP inhibitor, TRO40303 (Trophos), which ameliorated experimental alcoholic AP but has yet to be tested in clinical AP, was found to be ineffective in a clinical trial for myocardial infarction. Therefore there is a current, pressing need to identify and evaluate novel, potent and selective cyclophilin D inhibitors to protect against mitochondrial dysfunction in AP. At present, pharmaceutical companies and academic researchers are actively engaged in drug discovery programmes to address this important therapeutic opportunity.

In a timely new study in this issue of *The Journal of Physiology*, Tóth and colleagues (2019) have conducted a thorough investigation into the effects of the cyclophilin D inhibitor *N*‐methyl‐4‐isoleucine cyclosporine (NIM811) in experimental AP. Previously, beneficial actions of NIM811 have been reported in diverse pathologies, including ischaemia–reperfusion injury, central nervous system damage and encephalomyelitis. Since it exhibited no toxic immunosuppressant activity or serious adverse effects in an HCV‐infected patient study, potential clinical utility is indicated for diseases in which mitochondrial dysfunction is core. The exciting new results of Tóth *et al*. demonstrate promising protective actions of this compound in *in vivo* experimental AP models and *in vitro* isolated pancreatic cells. In ductal cells they found that NIM811 inhibited mitochondrial depolarisation and necrosis induced by bile acid and a fat/alcohol combination. Furthermore, cyclophilin D knockout (*ppif^−/−^*) was not only protective against mitochondrial dysfunction and necrotic cell death caused by AP precipitants in isolated pancreatic acinar cells, in accord with previous results (Mukherjee *et al*. 2016), but also in ductal cells, indicating a combined beneficial outcome in two cell types implicated in AP progression (Hegyi & Petersen, 2013). Importantly, oral treatment with NIM811, i.e. after AP induction, ameliorated both TLCS‐AP and FAEE‐AP *in vivo*, although its effects were noticeably weaker in the latter model, which may reflect mechanistic differences relating to aetiology and therefore have implications for therapy. No toxic effects of NIM811 administration *per se* were detected in the study and a fuller evaluation of its clinical potential to treat AP may be warranted.

## Additional information

### Competing interests

None declared.

### Author contributions

Sole author.

### Funding

None.
